# Supplementary material for: Growth-dependent concentration gradient of the oscillating Min system in Escherichia coli
Source: J Cell Biol. 2024 Dec 2;224(2):e202406107. doi: 10.1083/jcb.202406107 (PMC11613459; doi:10.1083/jcb.202406107)
Supplement: Table S1 — lists strains and plasmids. [file jcb_202406107_tables1.docx]

**Table S1**. List of strains and plasmids.

| **Strain** | **Genotype** | **Source** | **RRID** |
| --- | --- | --- | --- |
| DH5α | *ΔlacZ ΔM15 Δ(lacZYA-argF) U169 recA1 endA1 hsdR17(rK-mK+) supE44 thi-1 gyrA96 relA1* | (Taylor et al., 1993) | SCR_006368 |
| MC1000 | *araD139 Δ(araABC-leu)7679 galU galK Δ(lac)X74 rpsL thi* | (Casadaban and Cohen, 1980) | Addgene_71852 |
| BL21(DE3)/pLysS | *str. B F^–^ ompT gal dcm lon hsdSB(r_B_^–^ m_B_^–^*) *λ(DE3 [lacI lacUV5-T7_p07_ ind1 sam7 nin5]) [malB^+^]_K-12_(λS)* pLysS *[T7_p20_ ori_p15A_] cat* | (Studier and Moffatt, 1986) | SCR_012821 |
| W3110 | *F^-^ 𝜆^-^ IN(rrnD-rrnE)1 rph-1* | (Bachmann, 1972) | SCR_007682 |
| FW1541 | W3110, *ΔminD minE::sfgfp-minD minE kan frt* | (Wu et al., 2015b) | N/A |
| FW2454 | W3110, *hupA-mKO2::aph frt* | (Wu et al., 2015a) | N/A |
| SOT87 | W3110, *ΔminC minD minE* *cat frt* | This study | N/A |
| SOT88 | W3110, *ΔminC minD minE* *frt* | This study | N/A |
| Plasmid |  |  |  |
| pMLB1113 | *ColE1/pBR/pUC, bla* | (de Boer et al., 1989) | N/A |
| pET21a | f1, pBR322, P_T7_, *bla* | Novagen | Addgene_69745 |
| pKD3 | *oriR6Kγ, bla, rgnB, cat, FRT* | (Datsenko and Wanner, 2000) | Addgene_45605 |
| pKD46 | *oriR101, repA101(ts),* P*_ara_-gam-bet-exo, bla, araC, [tL3]* | (Datsenko and Wanner, 2000) | Addgene_45606 |
| pBVS4 | pVBS3, P*_lac_::sfgfp-minD minE frt, aph, bla* | (Wu et al., 2015b) | N/A |
| pEB2-mScarlet-I | *oripSC101,* P*_proC_:: mScarlet-I, aph* | (Balleza et al., 2018) | N/A |
| pdCas9-bacteria | p15A, P*_LtetO-1_*-*dcas9, cat* | (Qi et al., 2013) | Addgene_44249 |
| pJSB-*ftsZ^G55^- mCerulean - ftsZ^G56^* | pJSB, P*_ara_::ftsZ^G55^- mCerulean -ftsZ^Q56^, cat* | (Moore et al., 2017) | N/A |
| pYLS67 | pFX55, P*_lac_*::*cfp-minD minE-yfp*, *bla* | (Hsieh et al., 2010) | N/A |
| pFX55 | P*_lac_*::*minC minD minE-yfp*, *bla* | (Shih et al., 2002) | N/A |
| pSOT6 | pHTPP15, P*_T7_*::*trx-his_6x_ -minD*, *bla* | (Hsieh et al., 2010) | N/A |
| pSOT13 | pET21a, P*_T7_*::*minE-his_6x_*, *aph* | (Hsieh et al., 2010) | N/A |
| pSOT157 | pMLB1113, P*_lac_::ftsZ-yfp*, *bla* | This study | N/A |
| pSOT279 | pET21a, P*_lac_::his_6x_-sfgfp-minD, bla* | This study | N/A |
| pSOT291 | pMLB1113, P*_lac_::ftsZ-mKO2, bla* | This study | N/A |
| pSOT294 | pMLB1113, P*_lac_::ftsZ^G55^-mcerulean-ftsZ^Q56^, bla* | This study | N/A |
| pSOT295 | pMLB1113, P*_lac_::ftsZ^G55^-mKO2- ftsZ^Q56^, bla* | This study | N/A |
| pSOT329 | pMLB1113, P*_LtetO-1_::ftsZ^G55^-mKO2-ftsZ^Q56^, bla* | This study | N/A |
| pSOT370 | pMLB1113, P*_LtetO-1_::ftsA-mScarlet-I, bla* | This study | N/A |
