# Supplementary material for: Growth-dependent concentration gradient of the oscillating Min system in Escherichia coli
Source: J Cell Biol. 2024 Dec 2;224(2):e202406107. doi: 10.1083/jcb.202406107 (PMC11613459; doi:10.1083/jcb.202406107)
Supplement: Table S2 — lists oligonucleotides. [file jcb_202406107_tables2.docx]

**Table S2**. List of oligonucleotides. Underlined: primer sequence for the target gene; double underlined: primer sequence for the cloning vector; **boldface**: restriction site.

| **Primer** | **Sequence** | **Source** | **Purpose** |
| --- | --- | --- | --- |
| 910F | AACATCATCGCGCGCTGGCGATGATTAATAGCTAATTGAGTAAGGCCAGGGTGTAGGCTGGAGCTGCTTC | This study | SOT87 |
| 911R | CAAGGCAGAGATAACTCTGCCTTGAAGATAAATGCGCTTTTACAGCGGGCCATATGAATATCCTCCTTA | This study | SOT87 |
| 339F | AATT**CCCGGG**ATGTTTGAACCAATGGAACTTACC (XmaI) | This study | pSOT157 |
| 336R | CT**GGATCC**ATCAGCTTGCTTACGCAGGAAT (BamHI) | This study | pSOT157 |
| 439F | AA**GGATCC**ATCAGTAAAGGAG (BamHI) | This study | pSOT157 |
| 309R | AGTGCC**AAGCTT**ATTTGTAC (HindIII) | This study | pSOT157 |
| 279F | CTA**GCTAGC**ATGCACCACCACCACCACCACGGAGGAATCCGTAAAGGCGAAGAGCTG (NheI) | This study | pSOT279 |
| 279R | CGC**GGATCC**TCATCCTCCGAACAAGCGTTTGAG (BamHI) | This study | pSOT279 |
| 1297F | GAT**GGATCC**GTGAGTGTGAT (BamHI) | This study | pSOT291 |
| 1298R | GCC**AAGCTT**AGGAATGAGCT (HindIII) | This study | pSOT291 |
| 1299F | GAT**GGATCC**GTGAGTGTGATTAAACCAGAGA (BamHI) | This study | pSOT294 |
| 1300R | GCC**AAGCTT**AGGAATGAGCTACTGCATCTT (HindIII) | This study | pSOT294 |
| 1304F | CGC**CCCGGG**ATGTTTGAACCAATGG (XmaI) | This study | pSOT294 |
| 1305R | GCG**AAGCTT**TTAATCAGCTTGCTTA (HindIII) | This study | pSOT294 |
| 1307F | TTGGAGGATCCACC**CTCGAG**GTGAGTGTGATTAAACCAGAAATGAAG (XhoI) | This study | pSOT295 |
| 1308R | GTCTGGGTGGATCC**CTCGAG**GGAATGAGCTACTGCATCTTC (XhoI) | This study | pSOT295 |
| 1448F | GAGCGGATAACAATTTCACACAGGA | This study | pSOT329 |
| 1449R | ATCCGCTCATGAGACAATAACCCTG | This study | pSOT329 |
| 1450F | GTCTCATGAGCGGATACGTCTCATTTTCGC | This study | pSOT329 |
| 1451R | AATTGTTATCCGCTCCCATAGATCCTTTCT | This study | pSOT329 |
| 1563F | TGATTACGAATT**CCCGGG**ATGATCAAGGCGACGGAC (SmaI) | This study | pSOT370 |
| 1564R | AGAGCCGCCAGAGCCGCCTGAAAACTCTTTTCGCAGCC | This study | pSOT370 |
| 1565F | GGCTCTGGCGGCTCTATCAGTAAAGGAGAAGCTGTG | This study | pSOT370 |
| 1566R | CGGCCAGTGCC**AAGCTT**TTATTTGTATAGTTCATCC (HindIII) | This study | pSOT370 |
