# Supplementary material for: Growth-dependent concentration gradient of the oscillating Min system in Escherichia coli
Source: J Cell Biol. 2024 Dec 2;224(2):e202406107. doi: 10.1083/jcb.202406107 (PMC11613459; doi:10.1083/jcb.202406107)
Supplement: Table S3 — shows estimation of single-cell weight. [file jcb_202406107_tables3.docx]

**Table S3**. Estimation of single-cell weight ^a^.

|  | **FW1541** | **W3110** |
| --- | --- | --- |
| OD_600nm_ | 0.355−0.362 | 0.348−0.363 |
| Dry weight (mg/1 mL) | 0.112 | 0.104 |
| Wet weight ^b^ (mg/mL) | 0.448 | 0.416 |
| Cell count (CFU/mL) | 7.96×10^8^ | 7.71×10^8^ |
| Single-cell weight (g/cell) | 5.63×10^-13^ | 5.40×10^-13^ |

^a^ Sample repeats n≥3.

^b^ Estimated from the dry weight based on the assumption that water accounts for 75% of the cell weight (Bionumbers ID 105482).
