# Supplementary material for: Growth-dependent concentration gradient of the oscillating Min system in Escherichia coli
Source: J Cell Biol. 2024 Dec 2;224(2):e202406107. doi: 10.1083/jcb.202406107 (PMC11613459; doi:10.1083/jcb.202406107)
Supplement: Table S4 — shows comparison of parameters among different studies. [file jcb_202406107_tables4.docx]

**Table S4**. Comparison of parameters among different studies.

|  | Our model (#2827) | Meacci model ^a^ | Fischer model ^a^ | Wu model ^a^ |
| --- | --- | --- | --- | --- |
| Dimension  Kinetic  parameter ^b^ | 1 | 1 | 1 (Stochastic) | 3 |
| *k_D_* (1/s) ^c^ | 1.66 | －^f^ | 0.04 | 0.075 (μm/s) ^i^ |
| *k_dD_* (μm/s) | 0.22 | 0.00004 | －^h^ | 0.05 (μm^2^/s) ^i^ |
| *k_dE_* (μm/s) | 0.82 | 0.0003 | 0.14-0.54 (1/s) | 0.25 (μm^2^/s) ^i^ |
| *k_ADP→ATP_*  (1/s) | 1.09 | Infinite | Infinite | 1 |
| *k_de_* (1/s) ^d^ | 0.33 | 0.04 | 0.04 | 0.33 |
| *D_d_*, *D_de_* (μm^2^/s) ^d^ | 0.2 | 0.06 | 0.06 | 0.013 |
| *D_D_* (μm^2^/s) ^d^ | 16 | Infinite ^g^ | Infinite ^g^ | 16 |
| *D_E_* (μm^2^/s) ^d^ | 10 | Infinite ^g^ | Infinite ^g^ | 10 |
| MinD concentration ^e^  (µM; molecule*) | 1.95 (2205*)  ($c_{DD}+c_{DT}+ c_{d}+ c_{de}$) | 900* | 1800* | 0.85 |
| MinE concentration ^e^ (µM; molecule*) | 1.4 (1580*)  ($c_{E}+c_{de}$) | 350* | 675* | 0.31 |

^a^ Meacci model: ([Meacci and Kruse, 2005](#_ENREF_33)); Fischer model: ([Fischer-Friedrich et al., 2010](#_ENREF_14)); Wu model: (Wu et al., 2015a)

^b^ $k_{D}$: attachment of cytosolic MinD-ATP to the membrane; $k_{dD}$: MinD-ATP recruitment by the membrane-bound MinD-ATP; $k_{dE}$: recruitment of MinE to the membrane by the membrane-bound MinD-ATP; $k_{de}$: detachment of the MinDE complex from the membrane due to ATP hydrolysis in MinD by MinE stimulation; $k_{ADP\to ATP}$: recharging of MinD-ADP with ATP by nucleotide exchange; $D_{D}$: diffusion of cytosolic MinD-ADP and MinD-ATP, $D_{E}$: diffusion of cytosolic MinE; $D_{d}$: diffusion of the membrane-bound MinD-ATP; and $D_{de}$: diffusion of the membrane-bound MinD-MinE complex; $c_{DD}$: concentration of cytosolic MinD-ADP; $c_{DT}$: concentration of cytosolic MinD-ATP; $c_{d}$: concentration of membrane-bound MinD-ATP; $c_{de}$: concentration of the membrane-bound MinD-MinE complex; $c_{E}$: concentration of cytosolic MinE.

^c^ 0.0125 1/(μm s) from Fange and Elf, 2006 (Fange and Elf, 2006); 0.1 μm/s from Halatek and Frey 2012 (Halatek and Frey, 2012).

^d^ Fixed parameters in the initial screening process.

^e^ The indicated concentration is at a fixed cell length. Present work: 2.84 μm; Meacci model: 1 μm; Fischer model: 2.5 μm.

^f^ In Meacci model, there is no spontaneous attachment of MinD to the membrane. A maximum density of membrane-bound MinD was assumed as 1000 molecules/μm, and nonlinear fluxes accounting for the formation of MinD aggregates on membrane with additional parameters were included in the model.

^g^ These models assumed homogenous cytosolic distribution for MinD and MinE.

^h^ The Fischer model lacked $k_{dD}$ because of the assumption that membrane-bound MinD has a tendency to stick together due to random diffusion at the membrane.

^i^ Only 2D parameters are reported in Wu et al., 2015 (Wu et al., 2015b), which were stated to be converted to 3D parameters during simulation. A notable difference in the Wu model is the formation of reaction fluxes: these rate constants were multiplied by the concentration of cytosolic MinD (for $k_{D}$ and $k_{dD}$), or MinE (for $k_{dE}$) near the membrane. In contrast, in our 1D model, the total cytosolic MinD (MinE) concentration in the corresponding grid was used.
