# Supplementary material for: Growth-dependent concentration gradient of the oscillating Min system in Escherichia coli
Source: J Cell Biol. 2024 Dec 2;224(2):e202406107. doi: 10.1083/jcb.202406107 (PMC11613459; doi:10.1083/jcb.202406107)

Fig S1

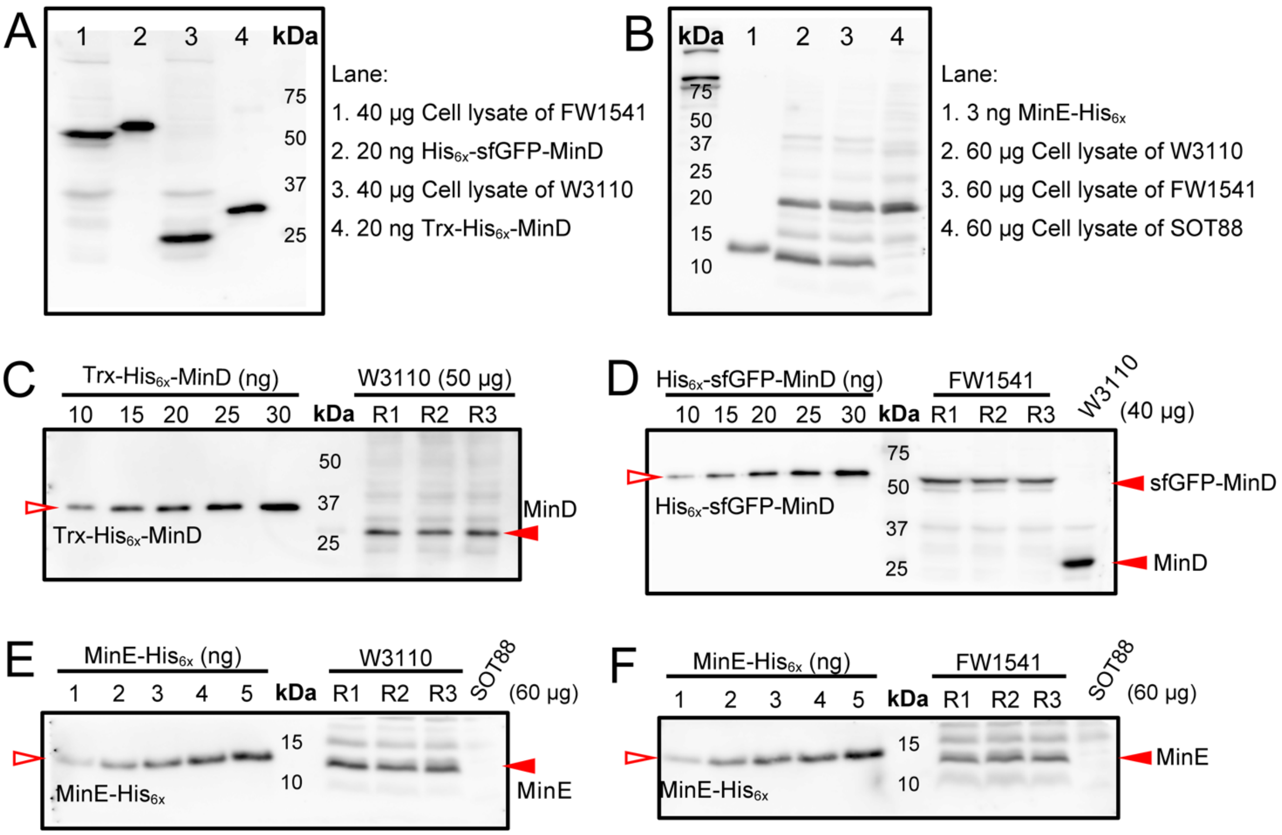
A
1 2 3 4 kDa
75
50
37
25
Lane:
1. 40 µg Cell lysate of FW1541
2. 20 ng His6x-sfGFP-MinD
3. 40 µg Cell lysate of W3110
4. 20 ng Trx-His6x-MinD
B
kDa 1 2 3 4
75
50
37
25
20
15
10
Lane:
1. 3 ng MinE-His6x
2. 60 µg Cell lysate of W3110
3. 60 µg Cell lysate of FW1541
4. 60 µg Cell lysate of SOT88
C
Trx-His6x-MinD (ng)
10 15 20 25 30
kDa
W3110 (50 µg)
R1 R2 R3
50
37
25
Trx-His6x-MinD
MinD
D
His6x-sfGFP-MinD (ng)
10 15 20 25 30
kDa
FW1541
R1 R2 R3
W3110
(40 µg)
75
50
37
25
His6x-sfGFP-MinD
sfGFP-MinD
MinD
E
MinE-His6x (ng)
1 2 3 4 5
kDa
W3110
R1 R2 R3
SOT88
(60 µg)
15
10
MinE-His6x
MinE
F
MinE-His6x (ng)
1 2 3 4 5
kDa
FW1541
R1 R2 R3
SOT88
(60 µg)
15
10
MinE-His6x
MinE

SourcedataFS#1A

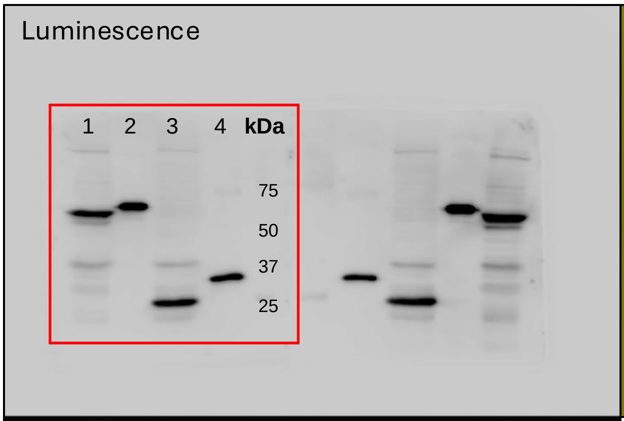

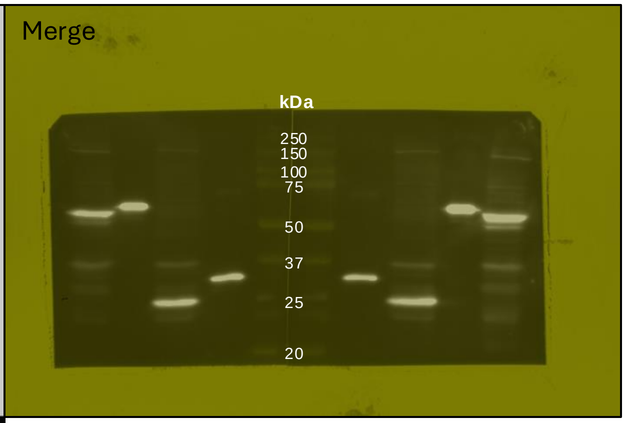

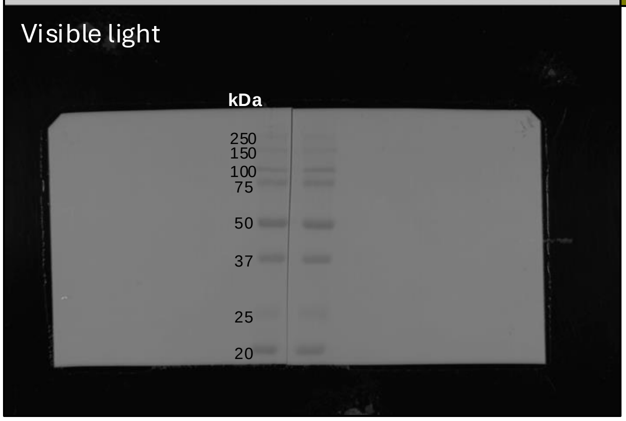

Lane:

1. 40 µg Cell lysate of FW1541
2. 20 ng His_{6x}-sfGFP-MinD
3. 40 µg Cell lysate of W3110
4. 20 ng Trx-His_{6x}-MinD

kDa (MW Marker)

SourcedataFS#1B

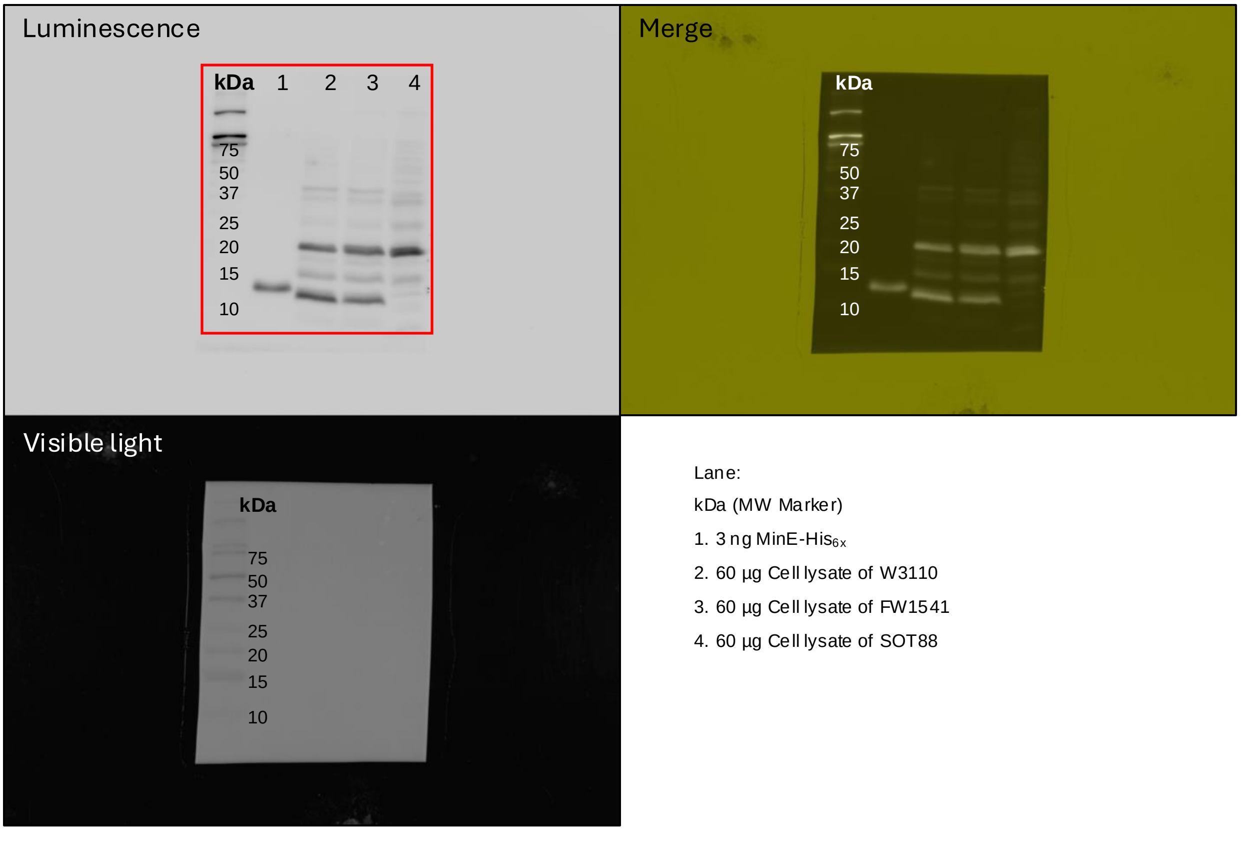

Lane:

kDa (MW Marker)

1. 3 ng MinE-His$_{6x}$
2. 60 µg Cell lysate of W3110
3. 60 µg Cell lysate of FW1541
4. 60 µg Cell lysate of SOT88

SourcedataFS#1C (Blot 1)

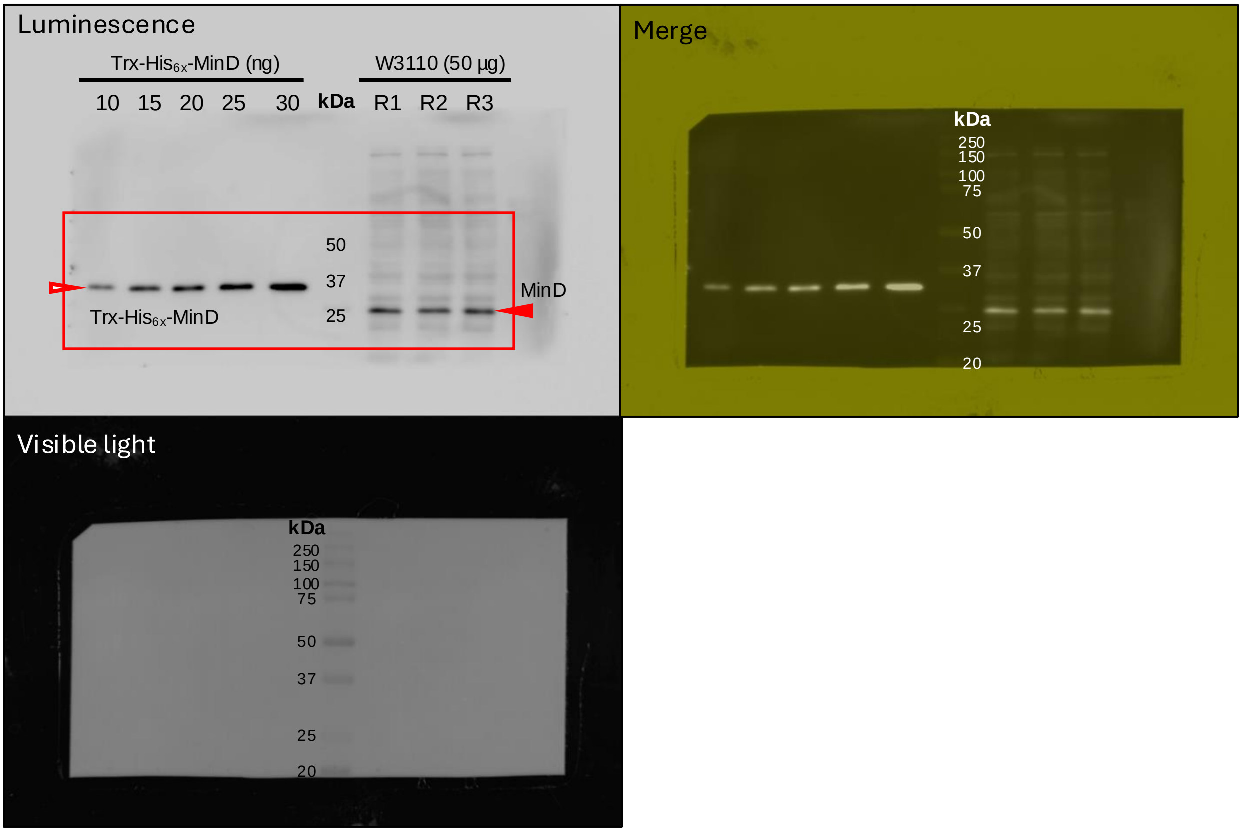

SourcedataFS#1C (Blot 2)

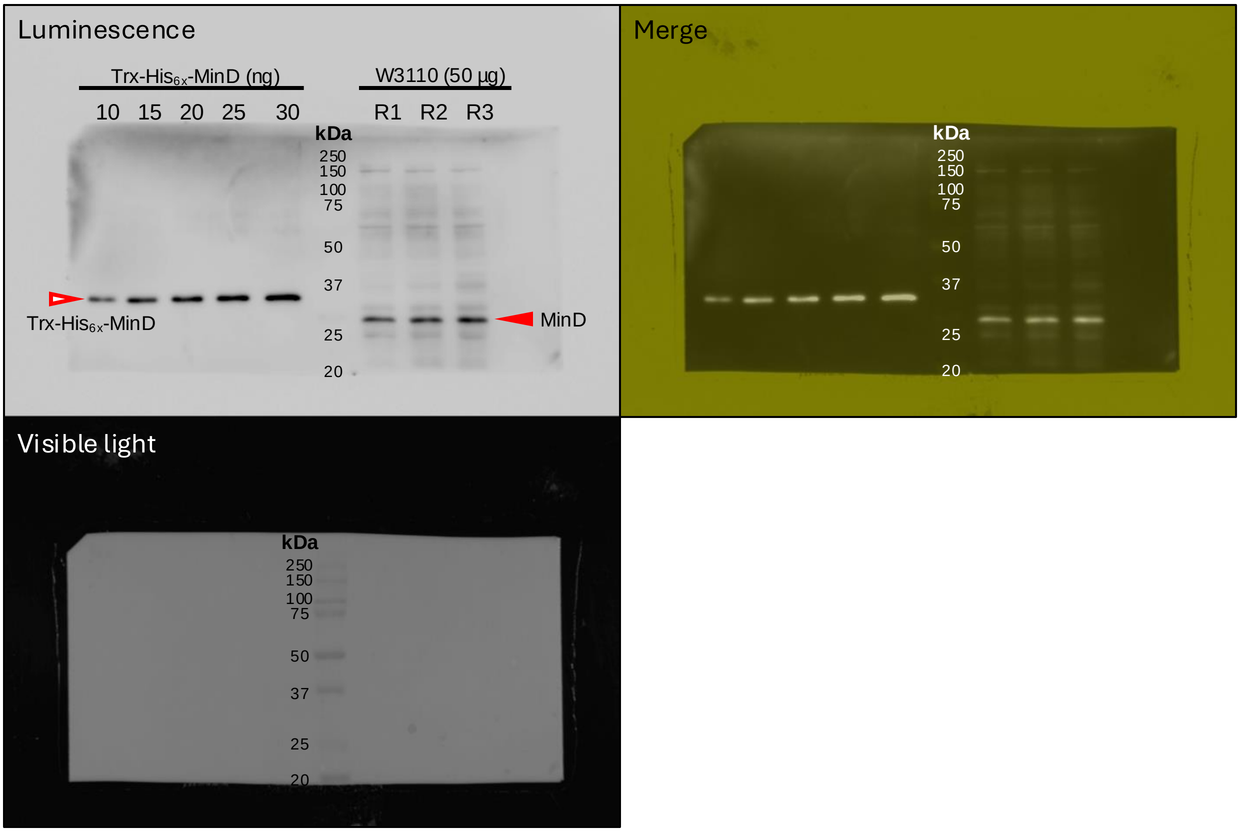

SourcedataFS#1D (Blot 1)

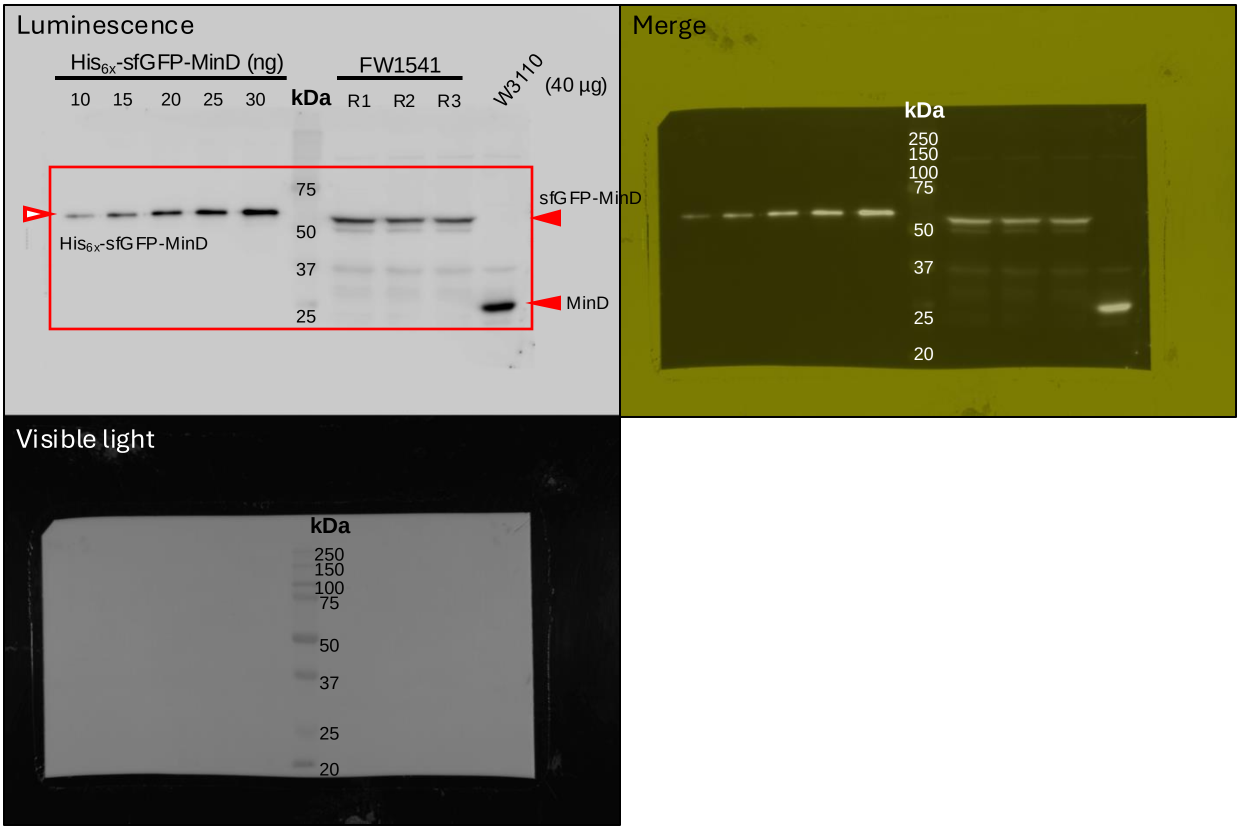

SourcedataFS#1D (Blot 2)

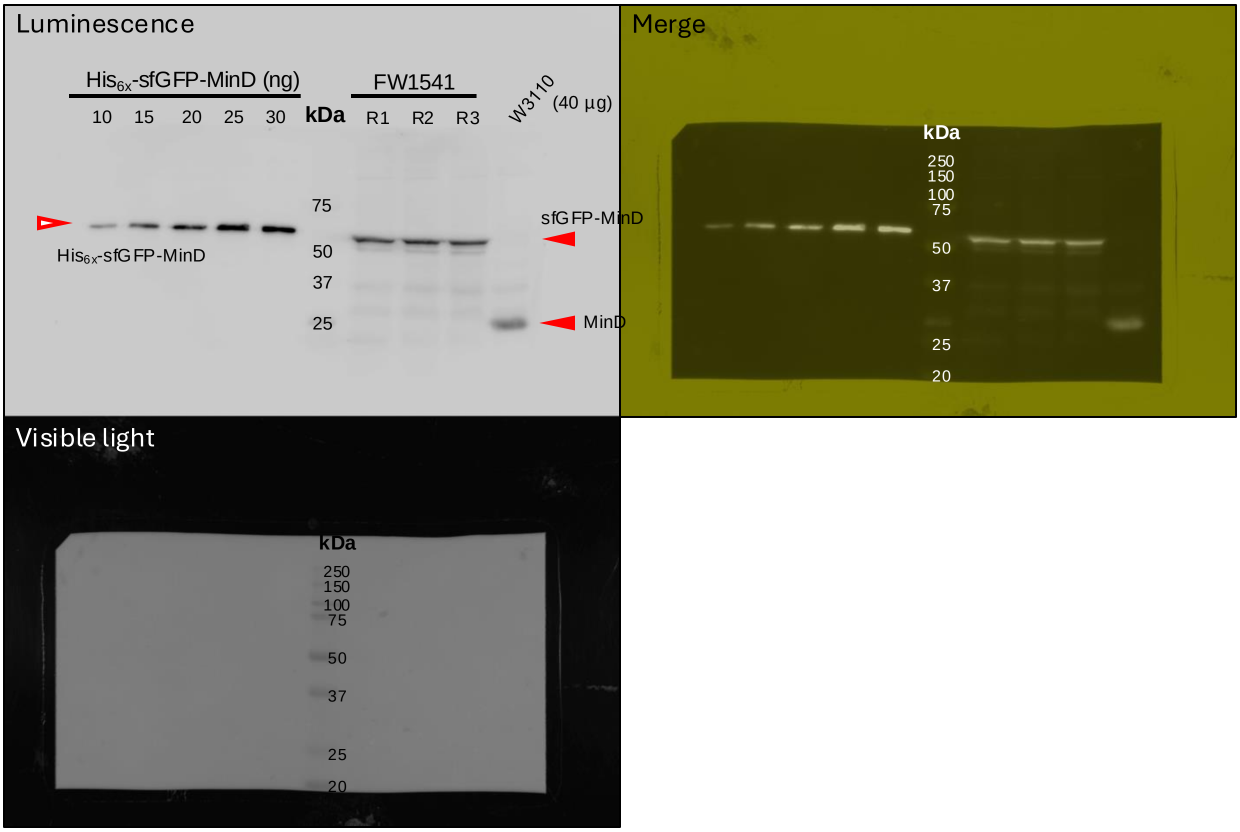

SourcedataFS#1D (Blot 3)

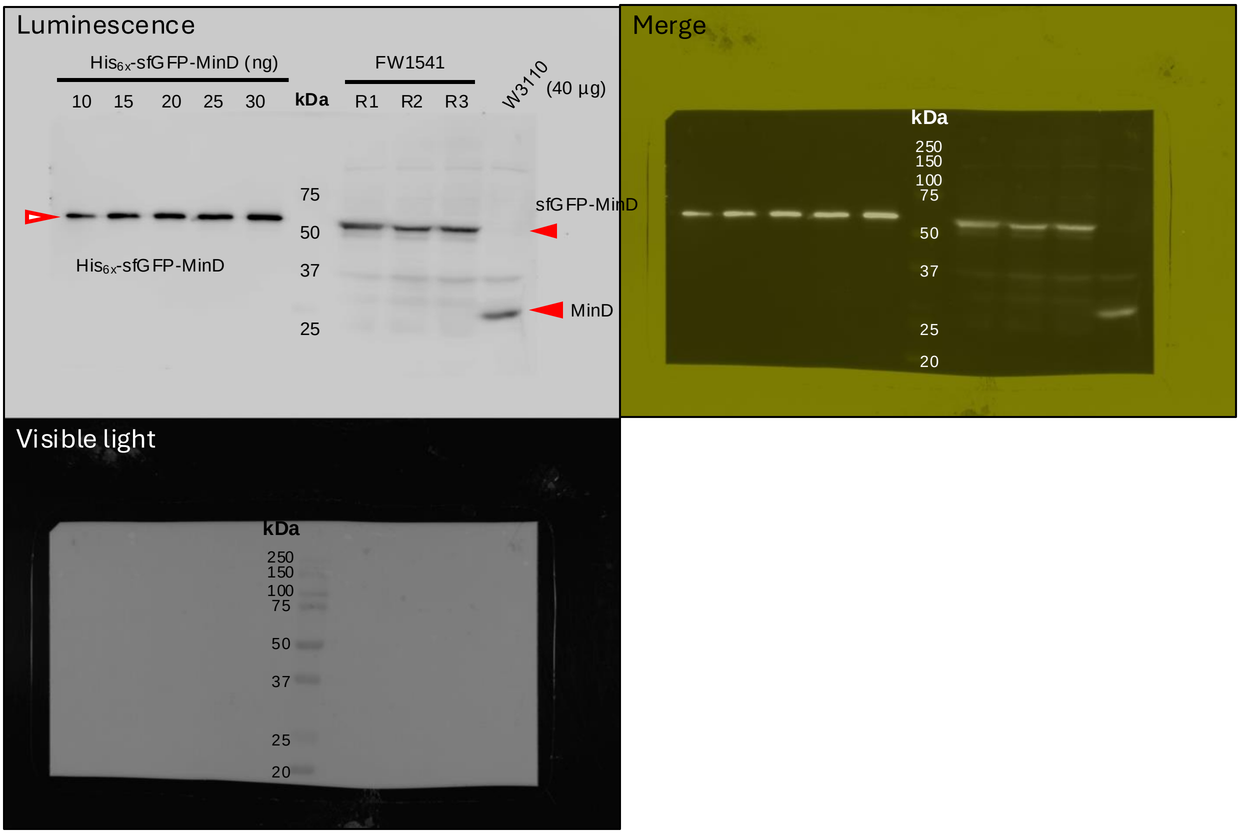

SourcedataFS#1E (Blot 1)

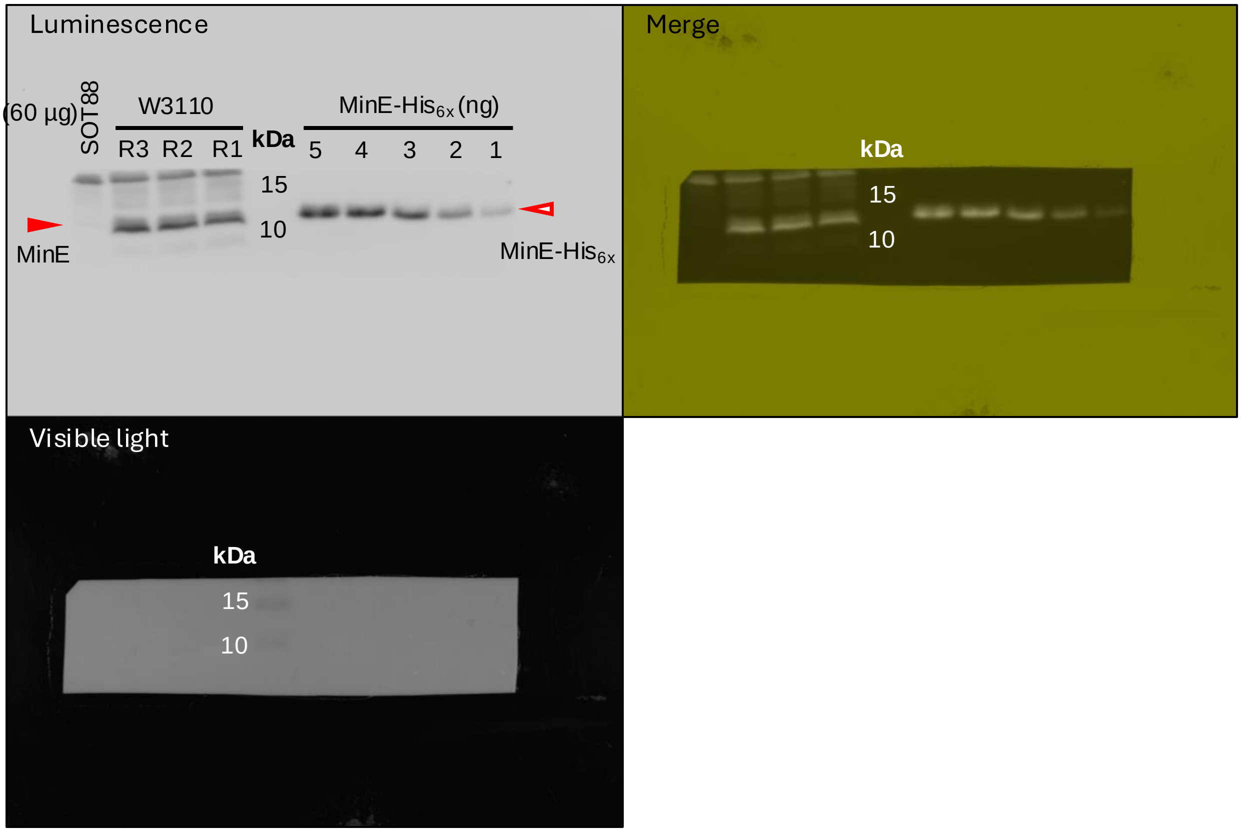

SourcedataFS#1E (Blot 2)

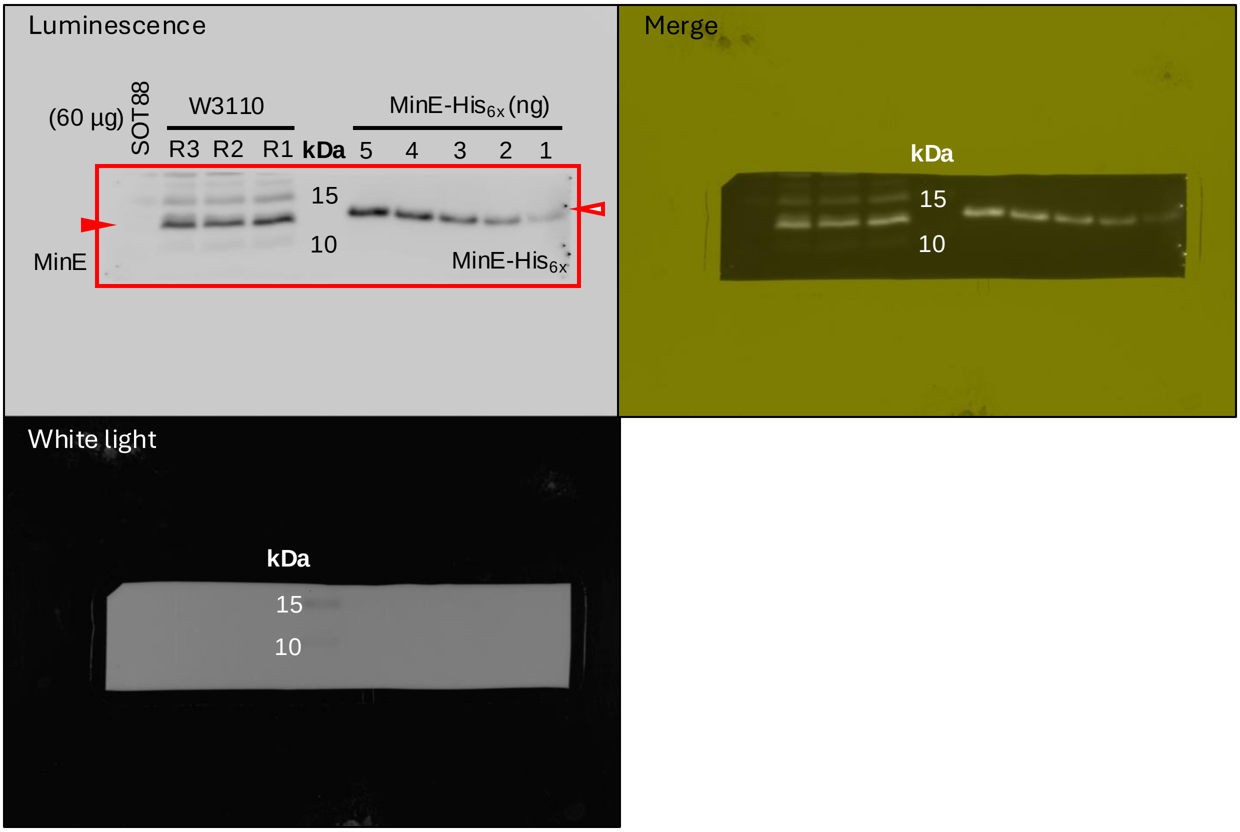

SourcedataFS#1E (Blot 3)

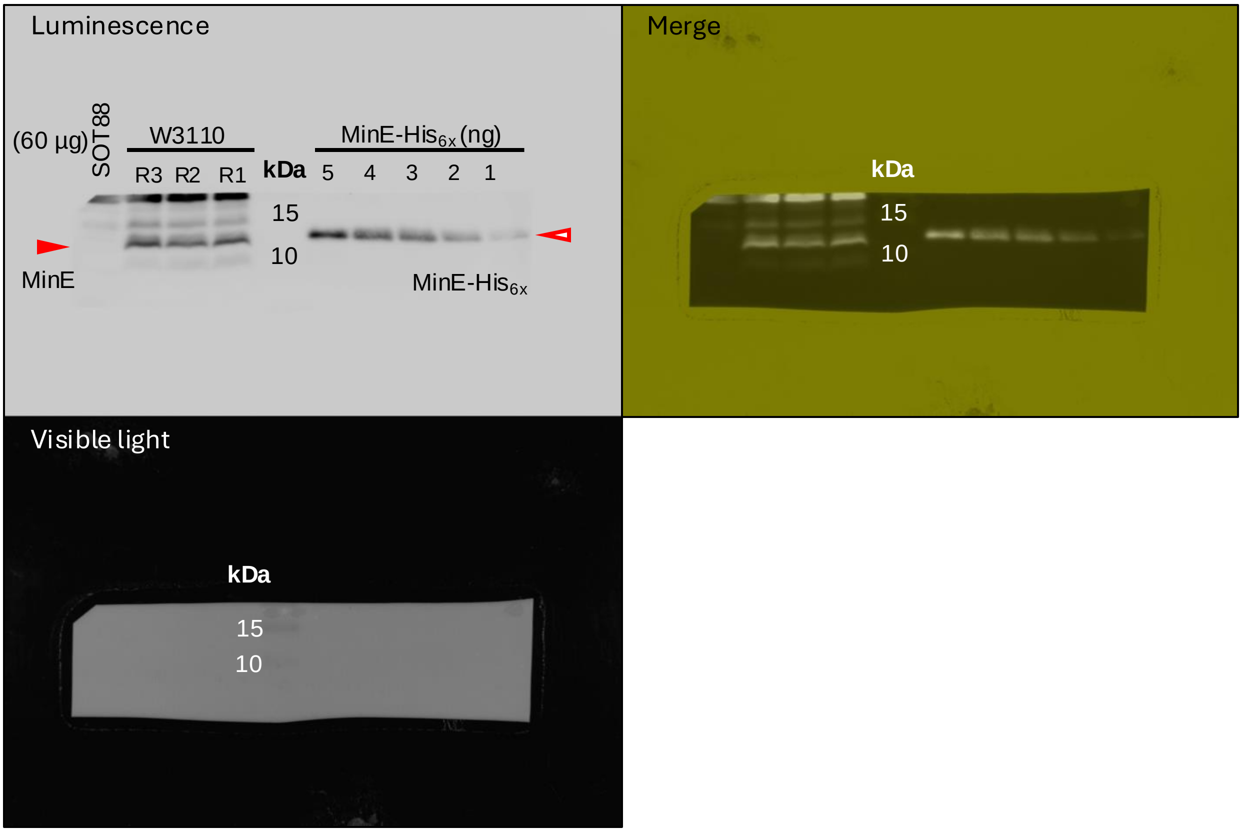

SourcedataFS#1E (Blot 4)

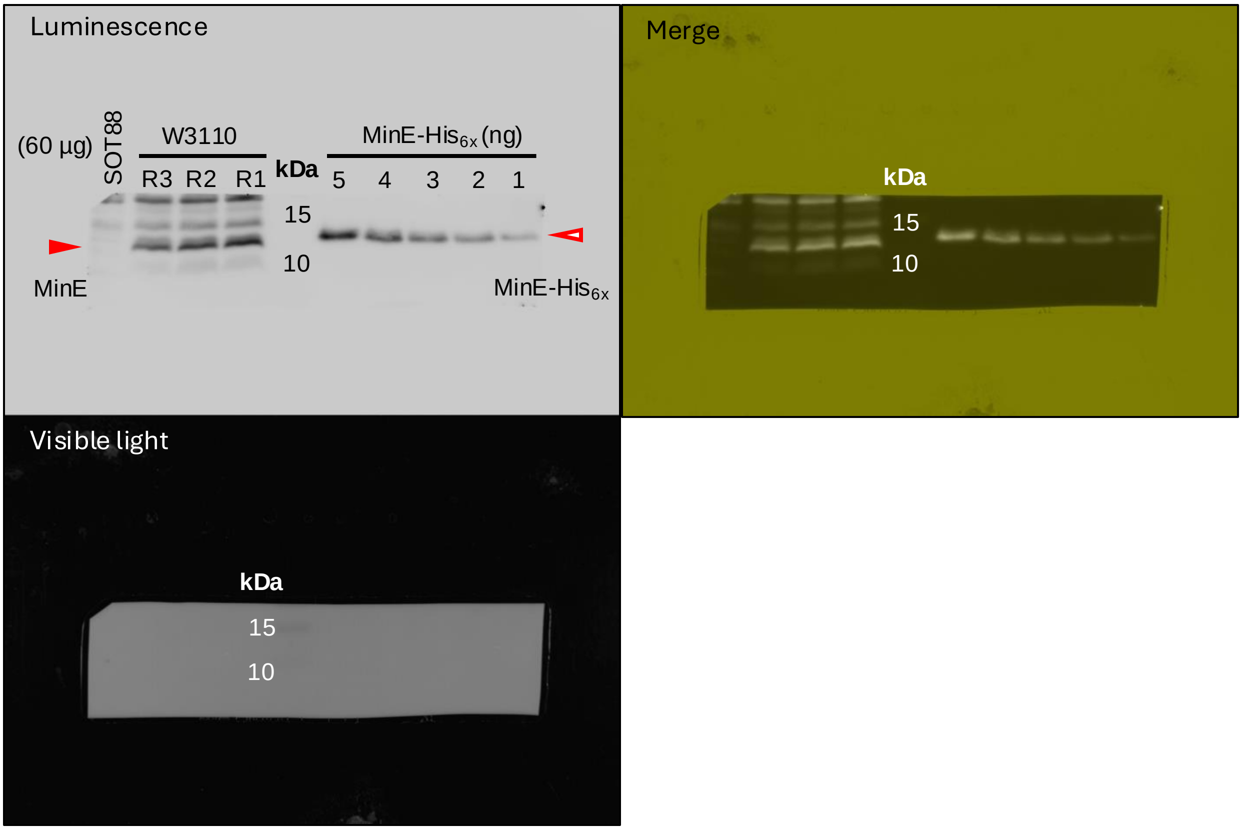

SourcedataFS#1E_blot 5

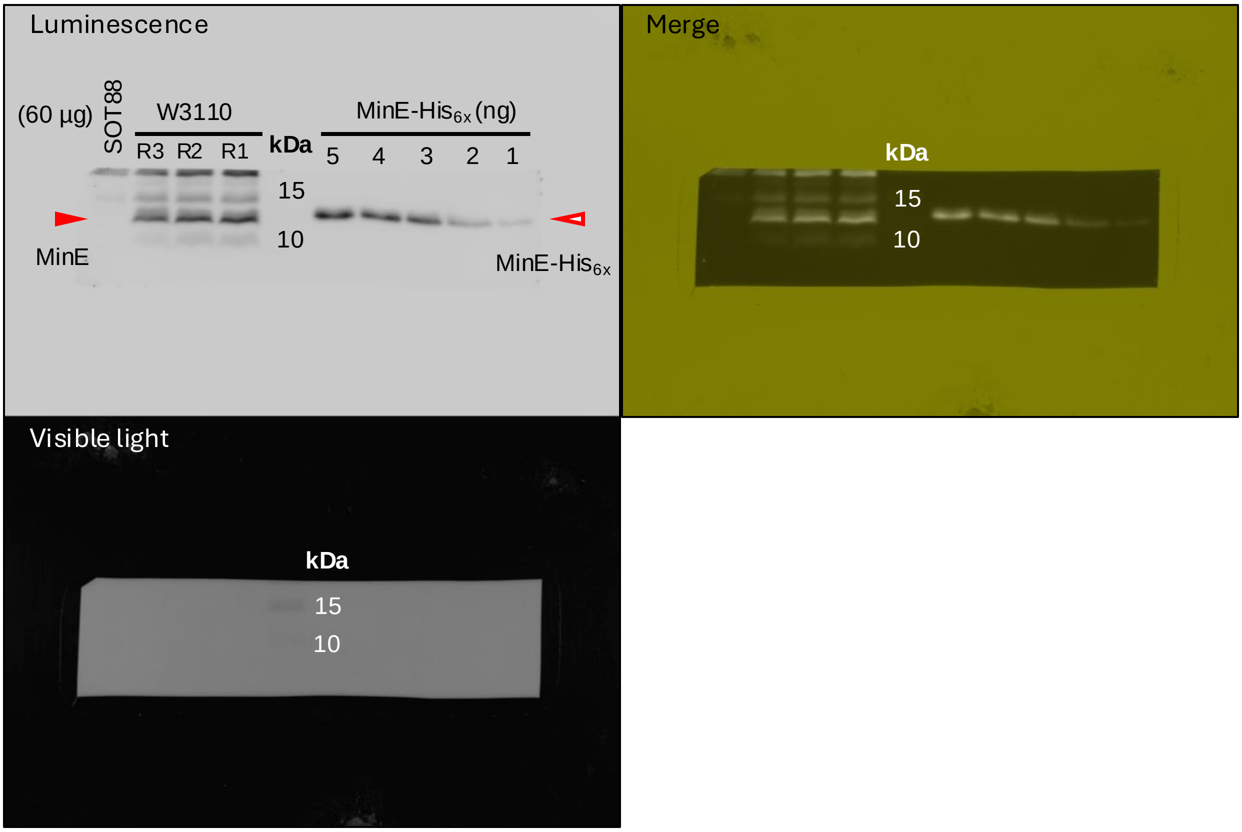

SourcedataFS#1F (Blot 1)

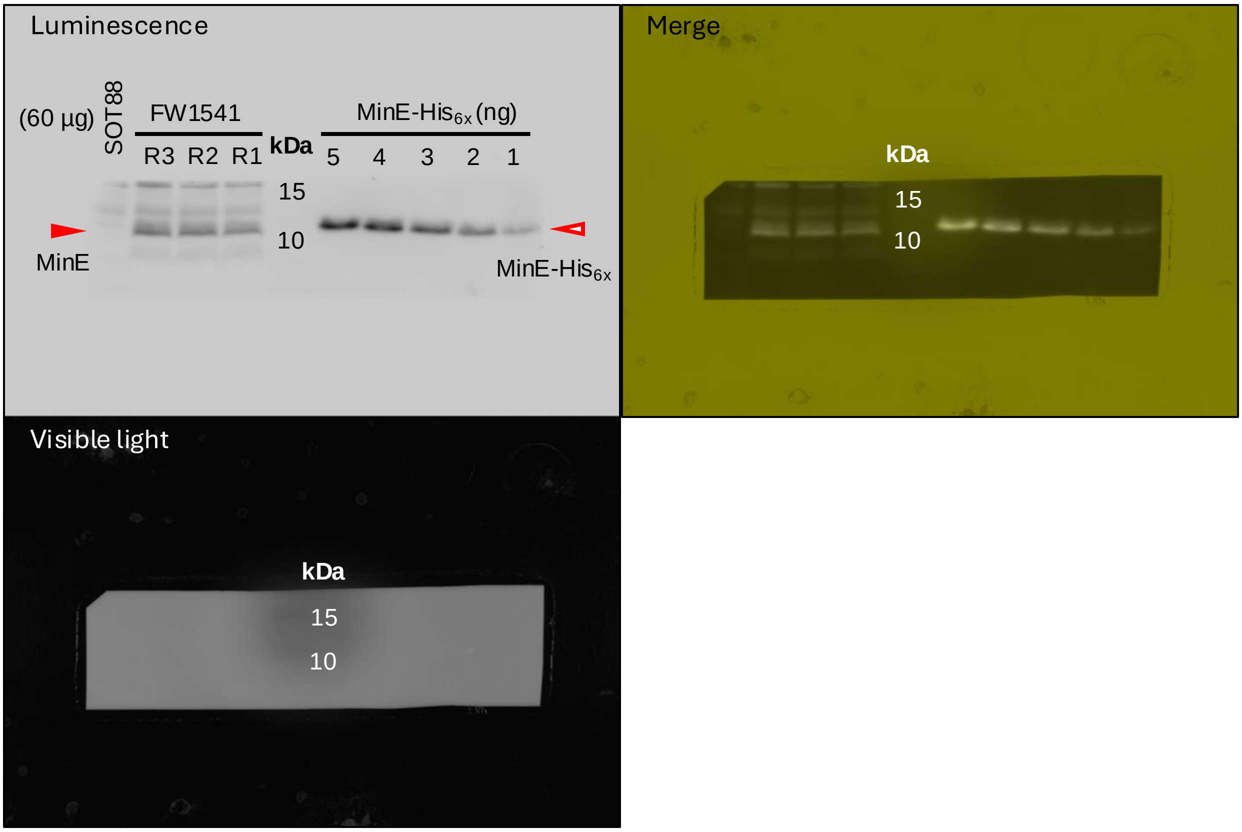

SourcedataFS#1F (Blot 2)

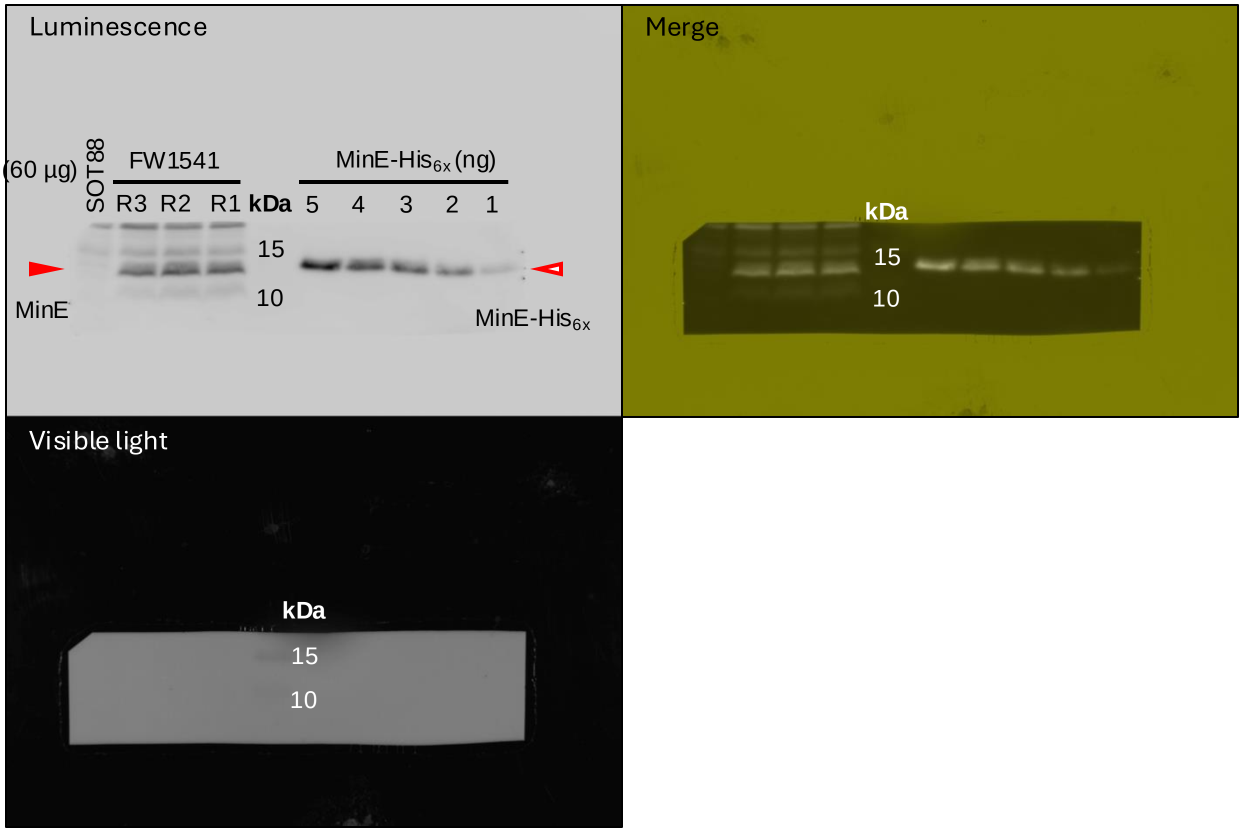

SourcedataFS#1F (Blot 3)

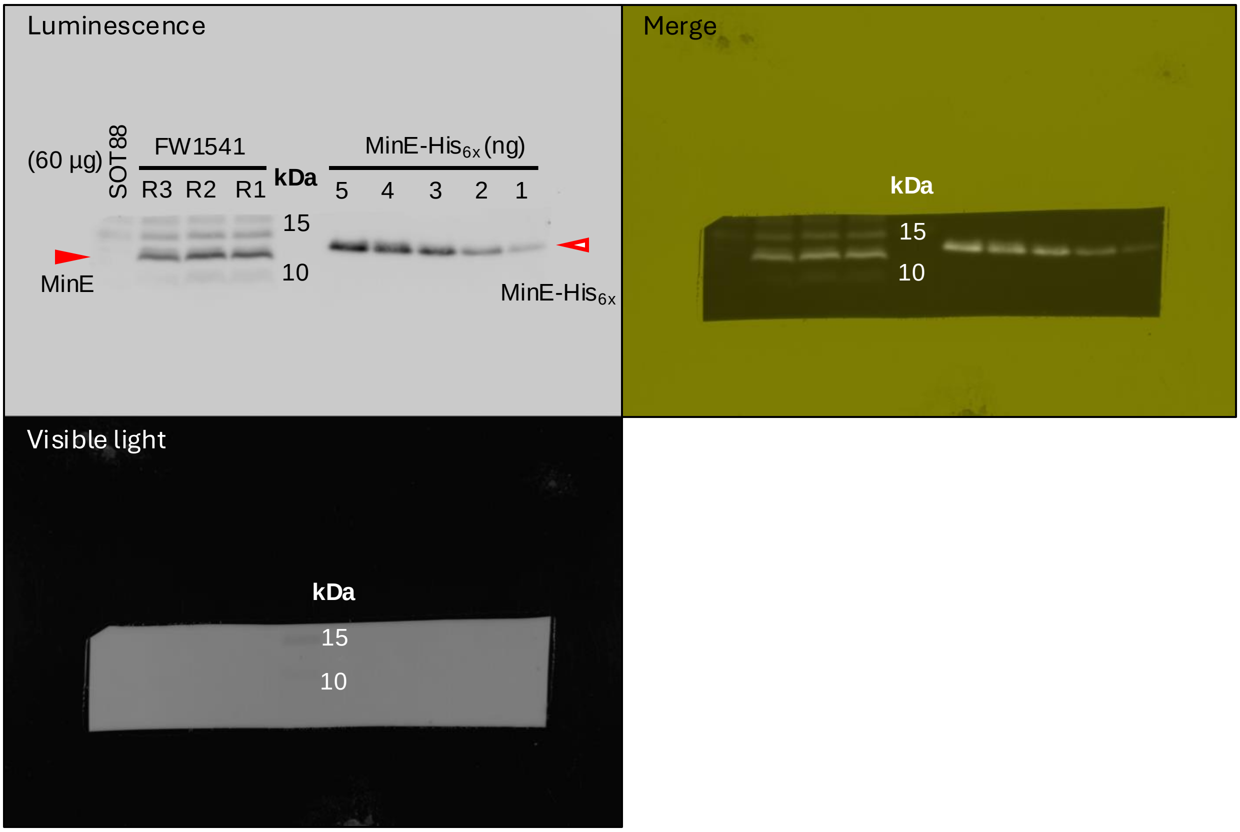

SourcedataFS#1F (Blot 4)

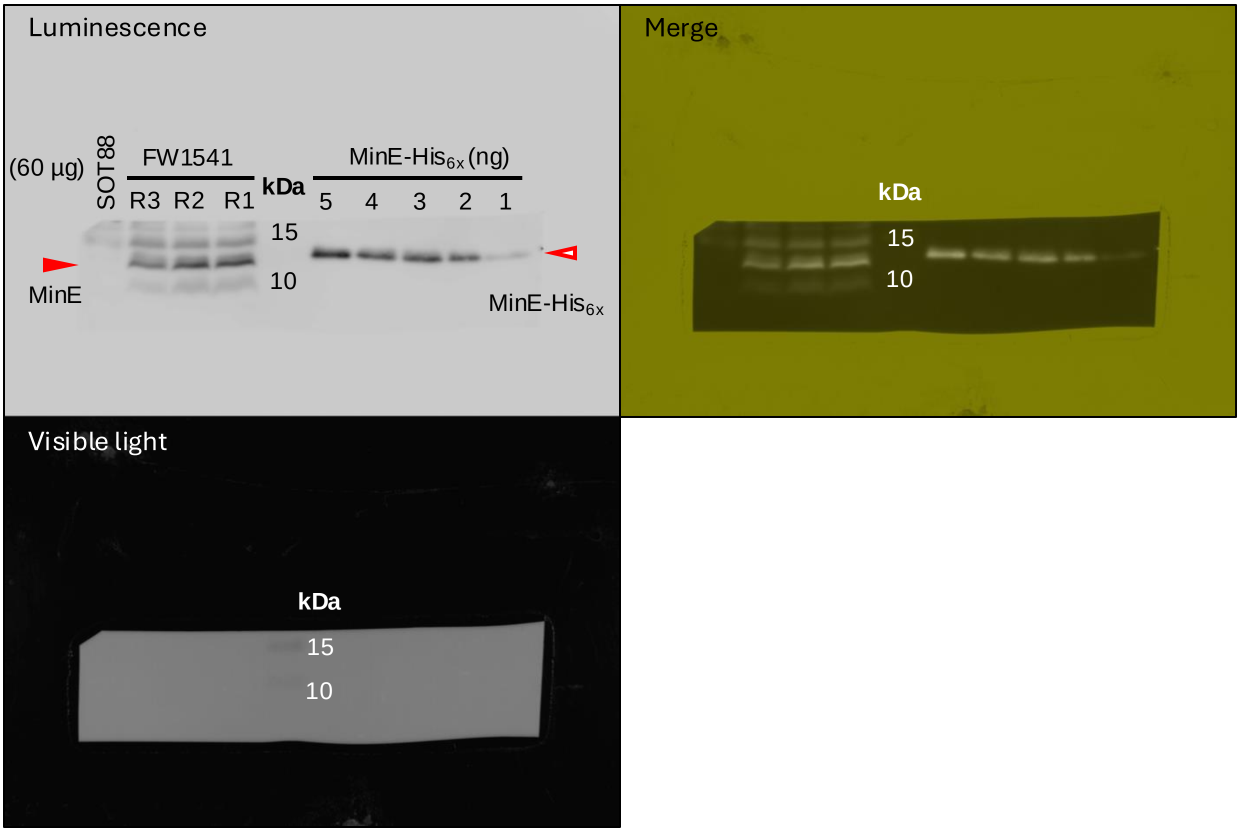

SourcedataFS#1F (Blot 5)

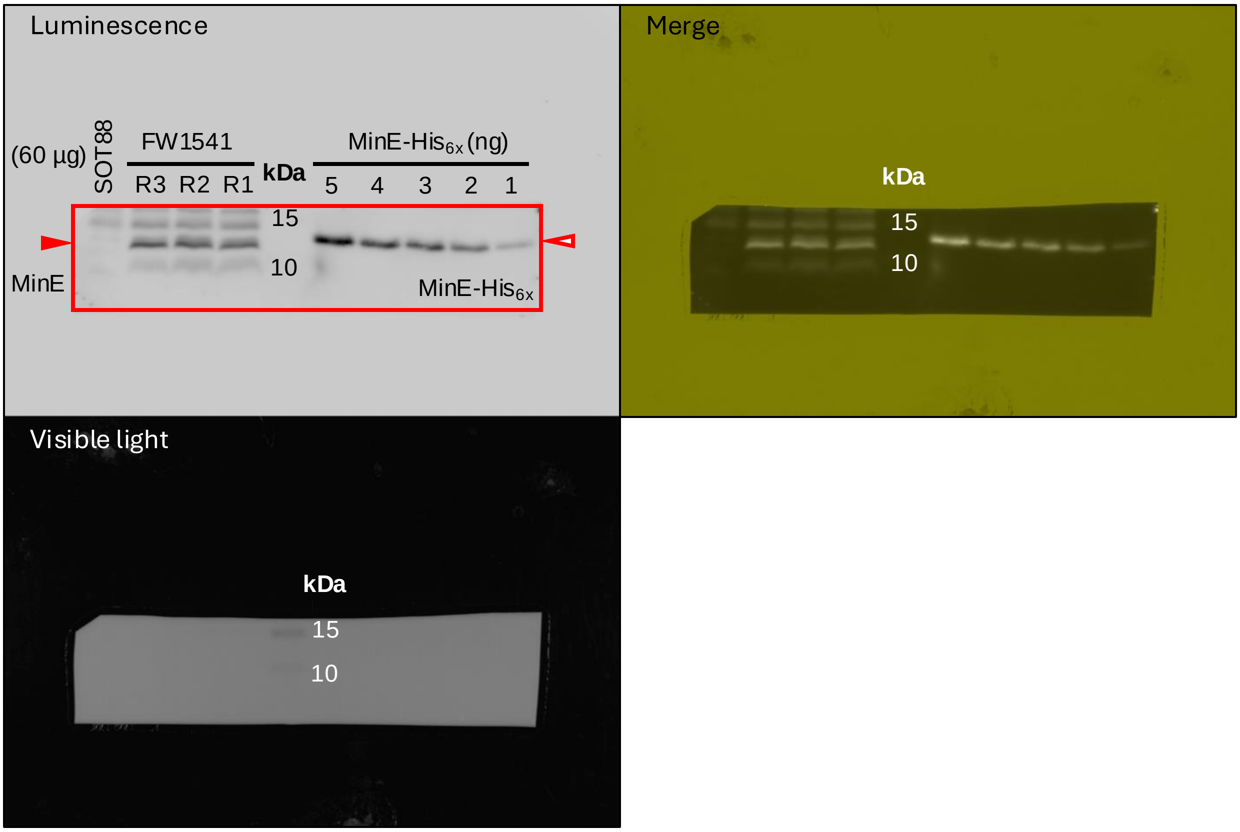

Supplement: SourceData FS1 — is the source file for Fig. S1. [file jcb_202406107_sourcedatafs1.pdf]
